# Vascular Surgery in Japan: 2016 Annual Report by the Japanese Society for Vascular Surgery

**DOI:** 10.3400/avd.ar.21-00110

**Published:** 2021-12-25

**Authors:** The Japanese Society for Vascular Surgery Database Management Committee Member, NCD Vascular Surgery Data Analysis Team

**Keywords:** peripheral arterial disease, stent graft, endovascular treatment, aneurysm, venous surgery

## Abstract

**Objectives**: This is an annual report indicating the number and early clinical results of annual vascular treatment performed by vascular surgeon in Japan in 2016, as analyzed by database management committee (DBC) members of the JSVS.

**Materials and Methods**: To survey the current status of vascular treatments performed by vascular surgeons in Japan, the DBC members of the JSVS analyzed the vascular treatment data provided by the National Clinical Database (NCD), including the number of treatments and early results such as operative and hospital mortality.

**Results**: In total 136,414 vascular treatments were registered by 1,070 institutions in 2016. This database is composed of 7 fields including treatment of aneurysms, chronic arterial occlusive disease, acute arterial occlusive disease, vascular injury, complication of previous vascular reconstruction, venous diseases, and other vascular treatments. The number of vascular treatments in each field was 21,653, 17,560, 4,983, 2,557, 846, 54,462 and 34,353, respectively. In the field of aneurysm treatment, 19,144 cases of abdominal aortic aneurysm (AAA) including common iliac aneurysm were registered, and 60.3% were treated by endovascular aneurysm repair (EVAR). Among AAA cases, 1,714 (9.4%) cases were registered as ruptured AAA. The operative mortality of ruptured and unruptured AAA was 15.7%, and 0.6%, respectively. 35.9% of ruptured AAA were treated by EVAR, and the EVAR ratio was gradually increasing, but the operative mortality of open repair and EVAR for ruptured AAA was 15.8%, and 15.3%, respectively. Regarding chronic arterial occlusive disease, open repair was performed in 9,303 cases, including 1,329 distal bypasses to the crural or pedal artery, whereas endovascular treatment (EVT) was performed in 8,257 cases. The EVT ratio was gradually increased at 47.0%. The number of varicose vein treatment tremendously increased to 52,639, and 68.5% of the cases were treated by endovenous laser ablations (EVLA). Regarding other vascular operations, 32,779 cases of vascular access operations and 1,411 lower limb amputation surgeries were included.

**Conclusions**: The number of vascular treatments increased since 2011, and the proportion of endovascular procedures increased in almost all field of vascular diseases, especially EVAR for AAA, EVT for chronic arterial occlusive disease, and EVLA for varicose veins. (This is a translation of Jpn J Vasc Surg 2021; 30: 23–41.)

## Introduction

Since the launch of the National Clinical Database (NCD) in 2011, the Japanese Society for Vascular Surgery (JSVS) has registered patients undergoing surgical procedures, including tabulated vascular surgical procedures among patients registered in the NCD, and released annual reports on vascular surgical procedures in scientific conferences.^1–9)^ This study reports the results of the tabulation of vascular surgical procedures registered in the NCD from January to December 2016 and the analysis by members of the JSVS database management committee.

## Methods

Vascular surgery data were extracted at the request of the JSVS, an NCD member association, from among surgeries registered in the NCD in 2016. Data were tabulated and classified into seven categories. The members of the database management committee of the JSVS verified the data and analyzed the tabulated results. The categories were as follows: 1) treatment for aneurysms, 2) revascularization for chronic arterial occlusions, 3) revascularization for acute arterial occlusions, 4) treatment for vascular trauma, 5) surgery for vascular complications after revascularization, 6) venous surgery, and 7) other vascular diseases and related surgeries.

The tabulation results presented include the number of patients who underwent differing surgical procedures, the etiology, operative mortality, in-hospital mortality, and materials used. Operative mortality is synonymous with surgery-related deaths and encompasses deaths within 30 days after surgery, including any deaths within 30 days after surgery regardless of cause or hospitalization status. In-hospital mortality refers to deaths that occurred during a period of continuous hospitalization after surgery, regardless of timing.

Although some numerical discrepancies exist in the tables presented, such as discrepancies in the sums under etiologies or materials used not being consistent with the total number of patients, the committee and the NCD carefully reviewed and concluded that the discrepancies were caused by one of the following four factors: 1) the selection of multiple choices, 2) blank entries when making selections, 3) omissions or erroneous entries by the party entering data, or 4) multiple types of materials being used or the treatment of multiple sites in a single surgical procedure. Since 2013, measures have been taken to prevent erroneous entries as much as possible by laying out or creating new options, and programs have been set up as much as possible for missing fields/entries and blocking registration for blank entries to prevent omissions.

Registration and tabulation methods that have changed since 2015 are presented in **Table 1**.

**Table:** Table 1 New items or changes in 2016 annual report

New items	Table number	Status until 2015
Lower limb artery		
Internal iliac	**Table 2-3**	Not existed

## Tabulation/Statistical Analysis Results

The total number of patients with vascular surgeries registered in the NCD in 2016 was 136,414 (a 9.7% increase over the figure in the previous year), exceeding 130,000. These constituted 9.0% of the total number of surgeries registered in the NCD in 2016. In addition, vascular surgeries were registered by 1,070 institutions, indicating that 28.2% of institutions registered vascular surgeries. Of these 1,070 institutions, 471 (44.0%) were certified as cardiovascular surgery training centers that contributed to data registration in 2016. Tabulation results for 2016 by category are presented below and analytical results are described. For statistical analyses using chi-square tests, a p-value of <0.05 was considered statistically significant.

## 1. Treatment for Aneurysms (Table 2-1 and 2-2)

### 1) Thoracic aortic aneurysms

Majority of thoracic aortic aneurysms are registered through the Japan Cardiovascular Surgery Database (JCVSD) by the JCVSD organization; however, some performed by vascular surgeons are tabulated in this vascular surgery database through the NCD (**Table 2**). Therefore, the registration of thoracic aortic aneurysm surgeries conducted across the entire country at present is fragmented, and it is not feasible to obtain an accurate image of the overall status. Thus, in the future, efforts should be made to obtain an overall view of thoracic aortic aneurysm surgeries performed nationwide through consultations with the JCVSD organization.

**Table:** Table 2 Treatment for aneurysm Table 2-1 Aortic aneurysm

Region of aortic aneurysm	Cases	Gender		Mortality		Ruptured aneurysm			Dissection^3)^	Etiology							
		Male	Female	30-day mortality	Hospital mortality	Cases	30-day mortality	Hospital mortality		Degenerative^4)^			Inflammatory	Vasculitis	Infected	Connective tissue disease^5)^	Others
										Cases	30-day mortality	Hospital mortality					
Ascending aorta^1)^	86	47	39	12	12	7	3	3	47	74	7	7	1	0	4	1	6
Aortic arch^1)^	598	469	129	24	34	44	8	8	219	522	22	31	2	0	8	26	40
Descending thoracic aorta^1)^	759	572	187	29	36	99	17	19	346	641	22	27	3	1	16	29	69
Thoracoabdominal aorta^1)^	419	324	95	25	37	41	13	18	125	379	22	32	4	0	13	6	17
Abdominal aortic aneurysm^2)^	19,144	15,766	3,378	393	505	1,794	282	344	779	18,404	356	444	235	11	275	30	189
with renal artery reconstruction	316	261	55	17	25	28	11	14	27	288	13	18	10	0	10	1	7
with renal artery clamping	1,363	1,145	218	47	69	178	36	45	70	1,272	40	55	36	0	35	1	19

1) These data are not including cases recorded in JCVSD Database in which most cardiac surgeons were entering their cases. 2) Including common iliac artery aneurysm. 3) Including both acute and chronic aortic dissection. 4) Most likely atherosclerosis. 5) Connective tissue abnormalities such as Marfan syndrome.

**Table:** Table 2-1 Aortic aneurysm (continued)

Region of aortic aneurysm	Treatment procedure						Graft materials^7)^		
	Replacement			Exclusion with bypass	Stent graft	Hybrid^6)^	Polyester	ePTFE	Others
	Cases	Y-graft	T-graft						
Ascending aorta^1)^	7	0	0	0	17	3	52	7	6
Aortic arch^1)^	20	0	0	2	411	130	90	94	10
Descending thoracic aorta^1)^	36	0	0	3	688	42	37	36	4
Thoracoabdominal aorta^1)^	51	0	0	11	279	19	103	20	4
Abdominal aortic aneurysm^2)^	7,691	5,700	1,018	60	11,507	40	6,765	364	77
with renal artery reconstruction	308	237	37	5	8	4	286	15	4
with renal artery clamping	1,354	1,061	210	10	8	3	1,283	58	11

6) Debranch bypass surgery combined with two staged TEVAR is counted as one case of hybrid treatment. 7) Only for open surgery.

**Table:** Table 2-2 Abdominal aortic aneurysm mortality classified by treatment procedures

Procedure for aneurysm repair	Ruptured aneurysm			Non-ruptured aneurysm		
	Cases	30-day mortality	Hospital mortality	Cases	30-day mortality	Hospital mortality
Replacement	1,146	181	220	6,458	53	79
Exclusion with bypass	21	2	4	39	1	2
EVAR^8)^	646	100	122	10,907	58	81
Hybrid	7	0	1	33	0	0

8) EVAR: endovascular aneurysm repair

### 2) Abdominal aortic aneurysms (Table 2-1 and 2-2)

**Figure 1:**
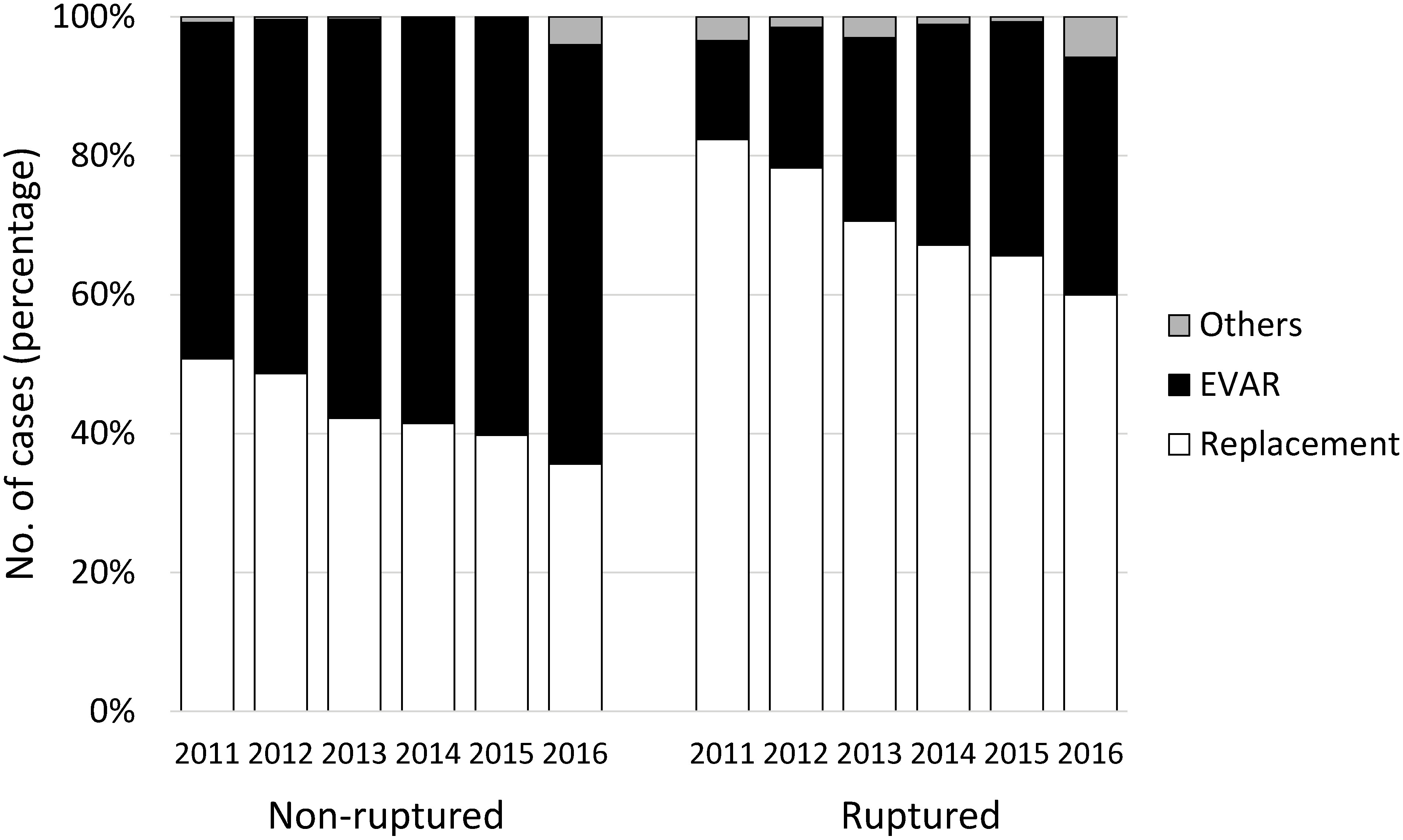
Fig. 1 Treatment procedure for non-ruptured and ruptured abdominal aortic aneurysm (AAA). Comparing year 2011, 2012, 2013, 2014 and 2015, proportion of EVAR selection was gradually increased in 2016.

The total number of patients who underwent surgeries for abdominal aortic aneurysms (including iliac artery aneurysms) registered in the NCD in 2016 was 19,144, and the number continued to increase by approximately 1,000 per year at 16,694 in 2013, 17,973 in 2014, and 18,907 in 2015; however, the increase was slight this year. Furthermore, 7,691 patients (40.2%) underwent replacement surgeries, and 11,547 patients (60.3%) underwent stent grafting (endovascular aneurysm repair [EVAR] including hybrid surgeries), and the proportions have been increasing even after EVAR took over the majority in 2013 (52.9% in 2013, 55.7% in 2014, and 57.6% in 2015) (**Fig. 1**). The number of replacement surgeries remains almost unchanged, ranging from 7,000 to 8,000.

Among those requiring replacement surgeries, renal artery clamping was required for 1,354 patients (17.6%) and renal artery reconstruction for 308 patients (4.0%). With the use of EVAR becoming more mainstream, the incidence of pararenal abdominal aortic aneurysms requiring renal artery clamping has been slightly increasing from 15.4% in 2013 to 15.8% in 2014 and 16.6% in 2015.

**Figure 2:**
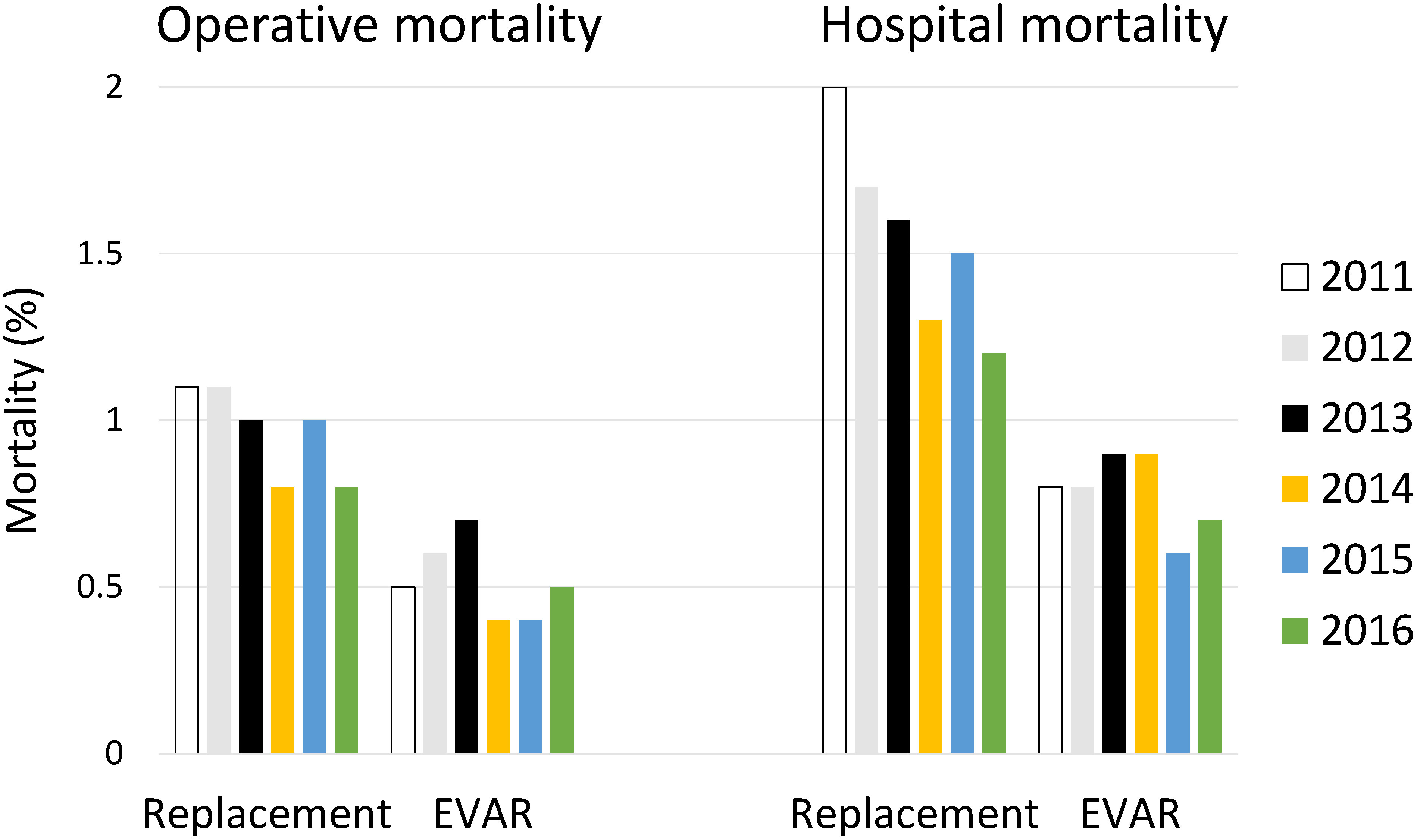
Fig. 2 Early clinical results of non-ruptured AAA in year 2016 comparing with those in year 2011, 2012, 2013, 2014 and 2015. Regarding the statistical difference of mortality rates between open repair (replacement) and EVAR, see main text. EVAR: endovascular aneurysm repair.

Although these were treatment results in nonruptured patients, the operative mortality for replacement surgeries was 0.8% and the in-hospital mortality was 1.2%; the rates were 0.5% (p=0.023) and 0.7% (p=0.002), respectively, for EVAR (including special and hybrid procedures) (**Fig. 2**). The rates worsened in patients who underwent replacement surgeries with the addition of renal artery clamping and were 0.9% and 2.0%, respectively, and were 2.0% and 3.8%, respectively, with the additional reconstruction.

**Figure 3:**
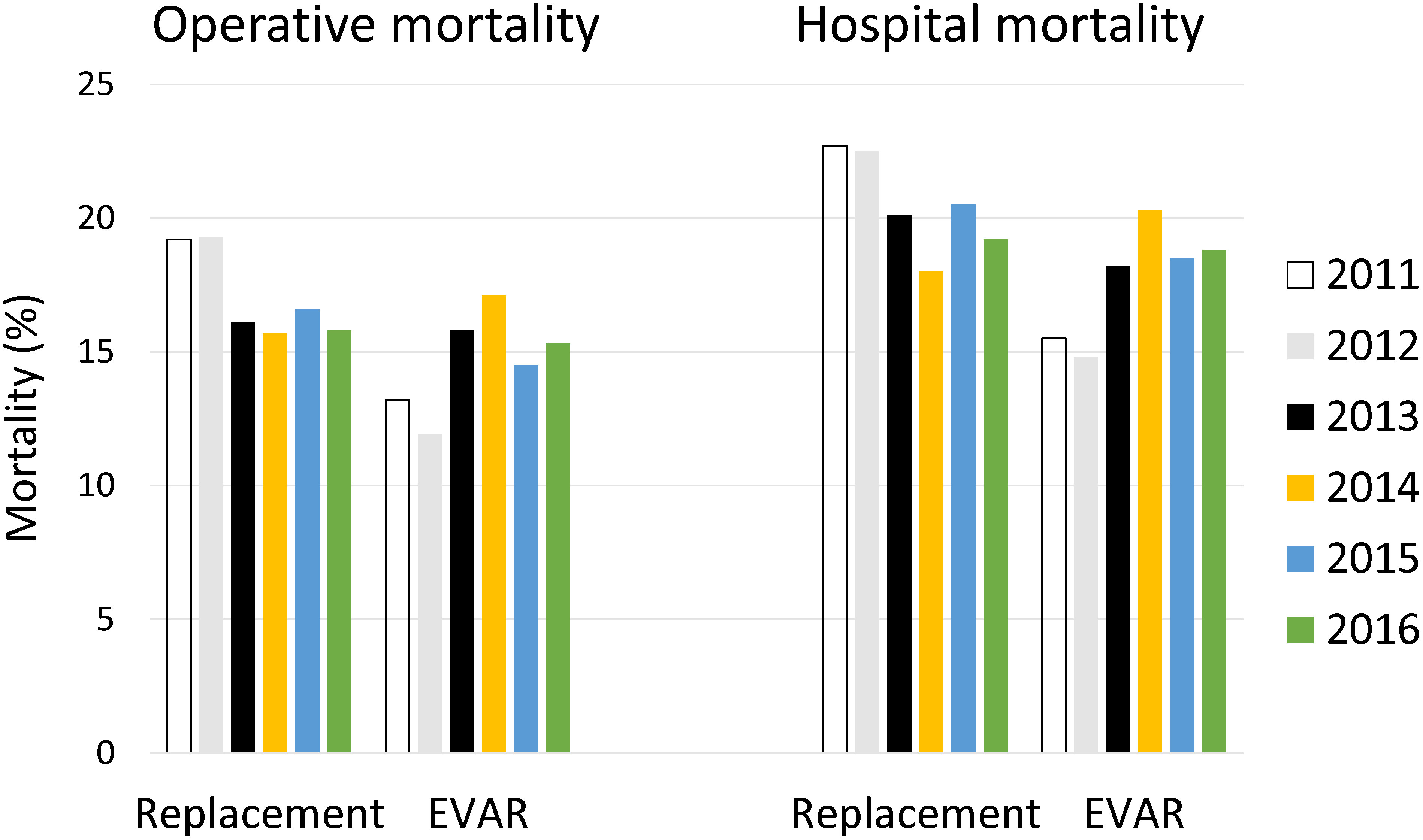
Fig. 3 Early clinical results of ruptured AAA in year 2016 comparing with those in year 2011, 2012, 2013, 2014 and 2015. Regarding the statistical difference of mortality rates between open repair (replacement) and EVAR, see main text.

Further, 1,794 patients had ruptures. The operative mortality was 15.7%, and in-hospital mortality was 19.2%. The results were similar to those in 2015 (16.0% and 19.9%, respectively). EVAR was performed in 653 patients (35.9%), and the proportion of patients with ruptures for whom EVAR is performed continues to increase (25.5% in 2013, 30.1% in 2014, and 33.9% in 2015) (**Fig. 1**). The operative mortality and in-hospital mortality for EVAR performed for patients with ruptures were 15.3% and 18.8%, respectively, which were almost the same as those in 2013 (15.8% and 18.2%, respectively), 2014 (17.1% and 20.3%, respectively), and 2015 (14.5% and 18.5%, respectively). Although there was a tendency for increases in EVAR performed for patients with ruptures, the results were stable. Even though there may be biases in the patient selection (EVAR may be selected for patients with general anatomical and hemodynamic advantages), the rates were no less than 15.8% and 19.2%, respectively, as seen for replacement surgery or slightly better (**Fig. 3**).

### 3) Peripheral artery aneurysms (Table 2-3)

The registration method was modified, and the item of internal iliac artery was added to the region of arteries of lower limbs. A total of 2,509 patients were registered; the male : female ratio was 1,843 : 666, with a higher incidence in males, and the breakdown of sites was 1,779 patients with sites in the lower limb arteries, 354 in the abdominal visceral arteries, 351 in the upper limb arteries, and 80 in the aortic arch branches. The inference was that 55 patients had simultaneous aneurysms at other sites. By artery, the breakdown was 42.5% for internal iliac arteries, 15.0% for femoral arteries, 9.5% for popliteal arteries, and 6.6% for brachial arteries. Details regarding internal iliac artery aneurysms were unilateral isolated in 41.5%, bilateral isolated in 14.0%, and simultaneous with abdominal aortic aneurysms in 37.2%. Symptoms were observed in 32.7%, a decrease of approximately 10% from the figure prior to 2015. Degenerative disease was the most common cause (75.5%). Coil embolization was used in 28.0% of the patients, stent grafts in 19.5%, ligation/resection in 19.8%, replacement surgery in 19.2%, and open bypass grafting in 5.3%, with endovascular treatment constituting 47.5%. The inference was that multiple surgical procedures were performed in 10.4% of the patients. The number registered and the incidence by site, etiology, symptomatic presentation, and proportion of endovascular treatment tended to differ from those prior to 2015; however, this was inferred to be associated with the aforementioned modification of registration method and seemed to more accurately show the actual clinical situation.

**Table:** Table 2-3 Peripheral artery aneurysm

Aneurysm	Cases	Gender		Mortality		Ruptured aneurysm			Etiology					Treatment procedure						Graft material for open surgery			
		Male	Female	30-day mortality	Hospital mortality	Cases	30-day mortality	Hospital mortality	Degenerative	Vasculitis^9)^	Infected	Traumas	Others	Replacement	Exclusion with bypass	Ligation/resection	Stent graft	Coil embolization	Others	Polyester	ePTFE	Autogenous vessel	Others
Aortic arch branches																							
Carotid	15	9	6	0	1	0	0	0	5	0	2	1	7	4	1	3	5	2	1	2	1	2	0
Vertebral	1	1	0	0	0	0	0	0	1	0	0	0	0	0	0	1	0	0	0	0	0	0	0
Subclavian	41	24	17	2	3	0	0	0	27	1	5	1	7	8	2	2	15	13	6	7	3	1	0
Multiple in arch branches	0	0	0	0	0	0	0	0	0	0	0	0	0	0	0	0	0	0	0	0	0	0	0
Others	23	13	10	2	3	0	0	0	7	1	4	1	10	4	1	8	8	4	3	4	1	0	0
Upper limb artery																							
Axillar	13	9	4	0	1	0	0	0	11	0	2	0	0	10	2	1	0	0	0	3	6	3	0
Brachial	170	95	75	1	3	2	0	0	34	2	21	44	69	35	8	84	1	1	48	6	11	24	1
Forearm-hand	128	74	54	1	1	2	0	0	45	1	17	33	32	14	2	94	0	0	25	2	8	4	1
Others	40	23	17	1	1	0	0	0	15	0	6	7	12	3	6	25	0	2	4	1	3	1	2
Visceral artery																							
Celiac	22	13	9	1	1	3	1	1	16	1	0	1	4	3	4	7	1	8	3	0	1	5	0
Hepatic	14	7	7	0	0	0	0	0	10	0	0	0	4	4	2	3	0	4	1	0	1	5	0
Splenic	81	35	46	0	0	1	0	0	73	1	1	1	5	1	4	13	0	61	5	0	0	4	0
Superior mesenteric	29	25	4	2	3	1	0	0	13	1	6	0	9	6	2	8	2	7	7	0	2	5	0
Renal	64	32	32	0	3	0	0	0	59	1	0	1	3	7	1	14	5	30	11	1	2	4	0
Others	144	110	34	2	3	2	0	0	118	0	7	1	18	25	12	17	52	55	6	27	6	3	0
Lower limb artery																							
Internal iliac	1,089	908	181	14	16	6	0	0	1,056	1	10	2	20	146	9	108	464	607	26	137	21	0	2
Femoral	384	290	94	10	18	2	0	0	186	1	39	56	102	142	18	142	13	10	79	61	85	19	2
Popliteal	244	176	68	2	3	0	0	0	222	0	1	6	15	143	77	31	0	1	7	20	83	117	0
Others	62	43	19	0	2	0	0	0	43	0	6	4	9	16	5	13	13	18	5	7	8	5	1
Total	2,509	1,843	666	37	60	18	1	1	1,894	10	126	159	320	550	153	568	558	802	231	268	234	197	9

9) Including TAO, Takayasu aortitis, collagen disease related vasculitis, Behcet disease, fibromuscular dysplasia. Abbreviations; Y-graft: Y-shape artificial graft; T-graft: straight artificial graft; Polyester: polyester artificial graft such as Dacron graft; ePTFE: expanded polytetrafluoroethylene graft

## 2. Revascularization for Chronic Arterial Occlusions (Table 3)

### 1) Arteries of arch branches, upper limbs, and abdominal viscera (Table 3-1)

The number of patients with carotid artery (288 patients), subclavian artery (580 patients), multiple aortic arch lesions (85 patients), and lesions in celiac and superior mesenteric arteries (124 patients) increased in 2016 compared with those in 2015. No significant changes were observed in other vertebral arteries and axillary arteries-arteries of upper limbs. The item of debranching associated with thoracic endovascular aortic repair (TEVAR/EVAR) was newly added in 2015, and the number of patients greatly differed from those prior to 2014. However, it is considered highly likely that the number of patients to be included in the item of debranching was also included in revascularization for occlusive disease this year. The above possibility is also strongly suggested by the number of patients who underwent carotid artery stenting (CAS) and carotid endarterectomy (CEA) for carotid arteries, remaining same as in 2015. Under TEVAR/EVAR-associated debranching in 2016, 34 patients were registered as undergoing ascending aorta-brachiocephalic artery-left common carotid artery (-left subclavian artery) bypasses; 284 patients underwent right axillary (subclavian artery)-left common carotid artery (-left subclavian artery) bypasses, right common carotid-left common carotid artery (-left subclavian artery) bypasses, or left common carotid-left subclavian artery bypasses; 325 patients underwent right axillary (subclavian)-left axillary (subclavian) artery bypasses; and 33 patients underwent abdominal aorta-superior mesenteric-renal artery bypasses. The number of bypasses significantly increased since 2015, with the exception of ascending aorta-brachiocephalic artery-left common carotid artery (-left subclavian artery) bypasses. Considering the increase in bypass surgeries thought to be associated with debranching mentioned above, the number of debranching associated with TEVAR seems to have substantially increased. Bypass surgeries for aortic arch branches thought to be related to these debranching procedures has been increasing year by year, which may be a manifestation of increased stent graft placement for anatomically complex aortic arch aneurysms (**Table 3-6**).

**Table:** Table 3 Reconstruction for chronic arterial occlusive diseases^10)^ Table 3-1 Arterial reconstruction for aortic arches

Aortic branches	Cases	Gender		Mortality	Background	Etiology					Revascularization procedures												Graft materials^14)^				Previous reconstruction					Revision reason							
		Male	Female	30-day mortality	Dialysis	ASO	TAO	Vasculitis^11)^	Takayasu arteritis	Others	CAS		CEA		PTA/stent^13)^	Replacement	Visceral artery bypass	Internal iliac artery bypass	Anatomical bypass	Carotid-subclavian bypass	Axillo-axillary bypass	Others	Polyester	ePTFE	Autogenous veins	Others	None	Once	Twice	Three times and more	Unclear	Host artery stenosis/occlusion	Graft stenosis	Graft occlusion	EVT stenosis	EVT occlusion	Stent graft-caused stenosis/occlusion	Poor symptom recovery	Other
											Cases	Brain complication^12)^	Cases	Brain complication^12)^	Cases																								
Carotid artery	289	228	61	17	20	67	0	0	2	4	11	0	50	4	17	3	0	0	22	175	134	30	73	153	9	4	279	8	2	0	0	3	3	1	2	0	0	0	3
Vertebral artery	14	11	3	1	1	1	0	0	0	4			0	0	1	0	0	0	1	6	7	6	5	5	2	0	13	1	0	0	0	0	0	0	0	0	0	0	1
Subclavian artery	580	448	132	16	37	87	0	3	6	26			0	0	75	3	1	0	25	243	344	63	155	379	4	4	558	18	1	2	1	5	2	4	3	3	1	0	3
Multiple lesions of arch branches	85	69	16	4	12	4	0	0	1	0			1	0	1	5	0	0	10	34	33	23	43	41	1	1	83	2	0	0	0	1	0	1	0	0	0	0	0
Upper limb including axillar artery	140	104	36	3	84	99	1	0	1	14			0	0	54	7	3	4	15	11	21	37	11	35	19	3	95	18	7	12	8	6	6	3	12	2	0	1	7
Celiac/Superior mesenteric artery	124	94	30	3	12	78	0	1	0	15			0	0	51	1	47	11	2	0	0	17	34	25	12	3	111	9	2	1	1	2	2	4	2	1	1	0	0
Renal artery	130	93	37	0	1	84	0	1	3	20			0	0	99	2	25	0	3	0	0	3	21	10	1	1	113	11	4	2	0	8	0	0	8	0	1	0	0
Others	0	0	0	0	0	0	0	0	0	0			0	0	0	0	0	0	0	0	0	0	0	0	0	0	0	0	0	0	0	0	0	0	0	0	0	0	0
Total	1,102	844	258	34	139	412	1	5	11	75	11	0	50	4	279	18	55	14	58	297	366	154	260	469	45	13	997	63	16	17	9	25	12	12	27	6	3	1	12

10) Bypass surgery combined with endovascular treatment is counted in both bypass category (**Table 3-2**) and endovascular category (**Table 3-5**). 11) Including TAO, Takayasu arteritis, Coarctation of aorta, collagen disease related vasculitis, Behcet disease, fibromuscular dysplasia. 12) Postoperative irreversible brain complication. 13) Including percutaneous transluminal angioplasty (PTA), stent, and other endovascular means such as catheter atherectomy. 14) Only for open surgery.

### 2) Anatomical bypass (Table 3-2), extra-anatomical bypass (Table 3-3), and endovascular treatment (Table 3-5) for the aorta to arteries of the lower limb region

**Figure 4:**
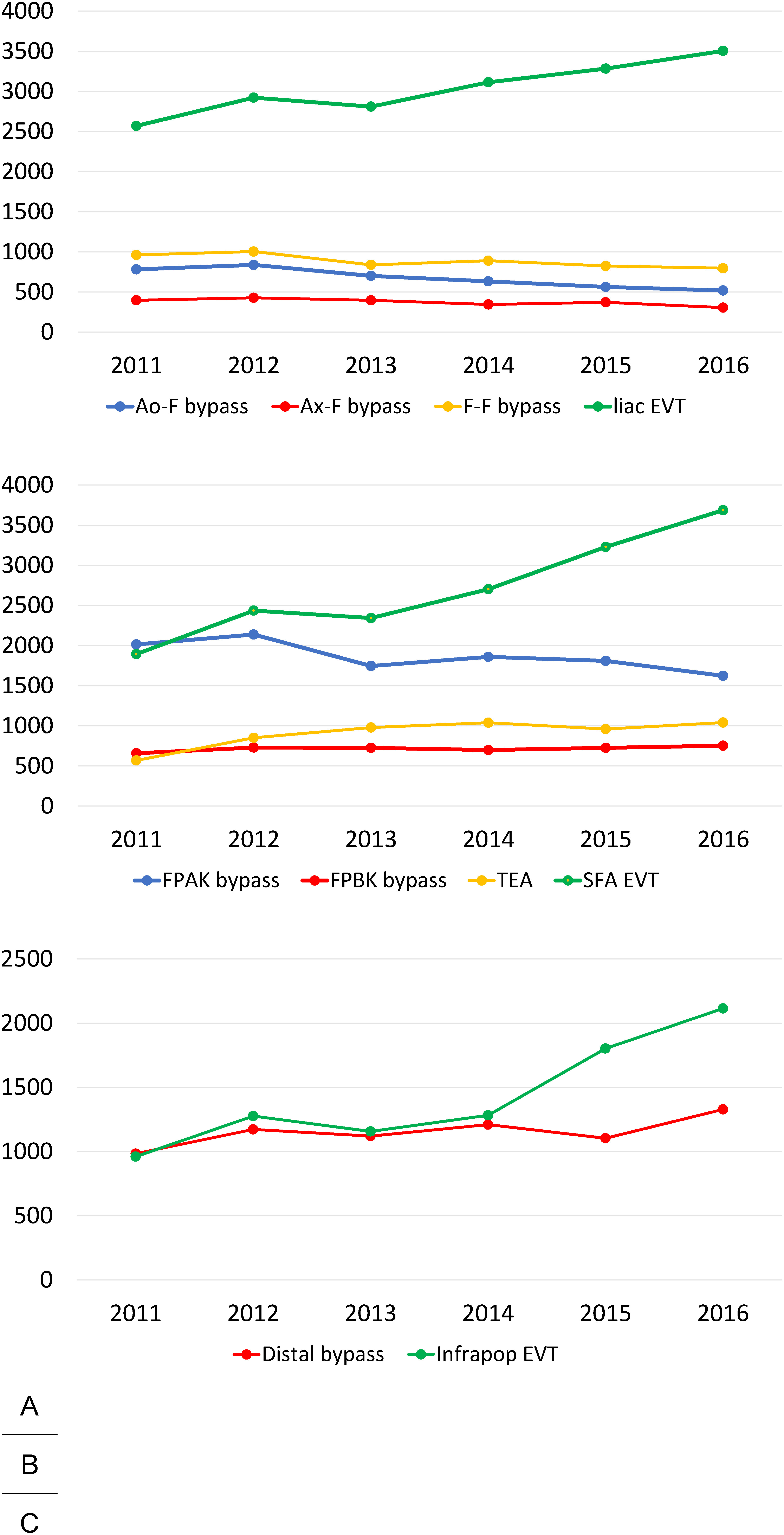
Fig. 4 The annual trends of the number of arterial reconstructions in aorto-iliac (**A**), femoro-popliteal (**B**), and crural/pedal region (**C**), comparing open repair and endovascular treatment.

**Aortic-iliac artery region**: The number of patients who underwent anatomical bypass surgeries for aortic-iliac artery region lesions was 640 in 2015 and 609 in 2016, with no changes in the breakdown, including the number of patients and graft materials used. Extra-anatomical revascularization procedures represented by axillary-femoral artery bypass and femoral-femoral artery bypass slightly decreased from 372 and 825 patients, respectively, in 2015 to 305 and 798 patients, respectively, in 2016, likely due to increases in endovascular treatment in this region (**Fig. 4A**). The rate of previous revascularization was 17% for anatomical bypasses versus 22% for extra-anatomical bypasses. The decrease in anatomical reconstruction was smaller than the increase in endovascular treatment, and the actual number of revascularization procedures in this region seemed to be slightly increasing.

**Table:** Table 3-2 Arterial reconstruction for chronic lower limb ischemia

From aorta to lower limb arterial systems	Cases	Gender		Mortality	Dialysis cases	Etiology					Graft materials				Previous reconstruction					Revision reason							
		Male	Female	30-day mortality		ASO	TAO	Vasculitis	Takayasu arteritis	Others	Polyester	ePTFE	Autogenous veins	Others	None	Once	Twice	Three times and more	Unclear	Host artery stenosis/occlusion	Graft stenosis	Graft occlusion	EVT stenosis	EVT occlusion	Stent graft-caused stenosis/occlusion	Poor symptom recovery	Other
Aorto-aortic bypass	52	35	17	1	7	43	0	1	1	3	30	17	6	2	40	8	2	2	0	1	3	5	1	0	1	2	1
Infrarenal aortic reconstruction (suprarenal clamp)	38	33	5	0	1	36	0	0	0	2	36	1	0	0	35	2	0	1	0	1	1	1	0	0	0	0	0
Aorto-femoral bypass^15)^	519	406	113	6	33	498	3	1	0	12	372	159	25	5	432	57	17	13	0	14	11	26	12	14	2	6	7
Femoro-popliteal (above the knee) bypass	1,624	1,201	423	26	265	1,606	3	2	2	11	244	1,129	338	24	1,166	283	94	71	10	103	24	102	40	81	11	58	40
Infrapopliteal arterial bypass	2,024	1,452	572	36	685	1,965	20	14	0	25	77	455	1,562	74	1,198	477	171	159	19	135	34	198	75	167	15	140	63
Femoro-popliteal (below the knee) bypass	752	522	230	17	186	733	3	3	0	13	41	341	422	25	462	172	55	55	8	42	17	93	21	51	7	39	24
Femoro-crural/pedal bypass^16)^	1,329	974	355	20	523	1,287	17	13	0	12	37	144	1,195	49	775	317	120	106	11	96	17	110	56	119	8	106	41
Others	111	87	24	2	26	105	1	0	0	3	44	53	25	1	66	23	9	12	1	17	4	11	7	2	0	3	2
Total	4,217	3,097	1,120	65	996	4,105	26	18	3	54	741	1,720	1,898	103	2,830	823	282	252	30	265	74	328	128	253	29	205	111

15) Including aorto-iliac bypass or ilio-femoral bypass. 16) Including popliteal-crural (or pedal) bypass.

**Table:** Table 3-3 Extra-anatomical bypass^17)^

Extra-anatomical bypass	Cases	Gender		Mortality	Dialysis cases	Etiology			Graft materials				Previous reconstruction					Revision reason							
		Male	Female	30-day mortality		ASO	TAO	Others	Polyester	ePTFE	Autogenous veins	Others	None	Once	Twice	Three times and more	Unclear	Host artery stenosis/occlusion	Graft stenosis	Graft occlusion	EVT stenosis	EVT occlusion	Stent graft-caused stenosis/occlusion	Poor symptom recovery	Other
Carotid-subclavian bypass	297	236	61	14	14	8	0	5	89	214	3	4	292	4	1	0	0	1	0	1	0	0	1	0	3
Axillo-axillary bypass	366	280	86	16	22	27	0	14	70	308	2	1	358	6	0	1	1	1	0	2	2	1	0	0	1
Axillo-femoral bypass^18)^	305	219	86	5	36	284	2	18	106	191	18	14	242	39	14	10	0	5	7	19	6	7	0	5	17
Femoro-femoral crossover bypass	798	635	163	8	72	758	1	33	203	569	56	20	619	130	24	22	3	27	7	51	14	26	11	22	21
Others	98	77	21	1	13	91	0	6	40	56	8	0	67	18	9	2	2	2	5	9	1	3	1	3	5
Total	1,697	1,320	377	36	144	1,145	2	74	463	1,206	84	37	1,415	193	48	35	6	35	19	81	23	37	13	29	46

17) Cases underwent extraanatomical bypass because of graft infection should not be included this category. Those cases are listed in vascular complication (**Table 6**). 18) A case underwent axillo-femoro-femoral crossover bypass is counted as one case. A case combined with additional contralateral side of axillo-femoral bypass as second staged surgery is counted as 2 cases.

**Table:** Table 3-4 Thromboendarterectomy^19)^ for chronic lower limb ischemia

Thromboendarterectomy	Cases	Gender		Mortality	Dialysis cases	Etiology			Previous reconstruction					Revision reason							
		Male	Female	30-day mortality		ASO	TAO	Others	None	Once	Twice	Three times and more	Unclear	Host artery stenosis/occlusion	Graft stenosis	Graft occlusion	EVT stenosis	EVT occlusion	Stent graft-caused stenosis/occlusion	Poor symptom recovery	Other
Aorto-iliac lesion	62	42	20	2	13	60	0	1	54	7	1	0	0	2	2	0	0	3	0	2	0
Femoro-popliteal lesion	1,040	744	296	11	278	1,021	15	4	800	162	45	27	6	90	14	23	34	23	1	35	29
Others^20)^	546	411	135	16	140	510	5	27	314	132	44	54	2	33	45	31	14	22	3	21	64
Total	1,611	1,169	442	28	415	1,554	20	32	1,146	291	87	79	8	119	58	54	47	46	4	57	90

19) Including patch plasty. 20) Including reconstruction, thrombolysis and others.

**Table:** Table 3-5 Endovascular treatment for chronic lower limb ischemia^13)^

Endovascular treatment	Cases	Gender		Mortality		Dialysis cases	Etiology			Previous reconstruction					Revision reason							
		Male	Female	30-day mortality	Hospital mortality		ASO	TAO	Others	None	Once	Twice	Three times and more	Unclear	Host artery stenosis/occlusion	Graft stenosis	Graft occlusion	EVT stenosis	EVT occlusion	Stent graft-caused stenosis/occlusion	Poor symptom recovery	Other
Aorto-iliac lesion^21)^	3,504	2,812	692	26	56	476	3,430	1	52	2,759	463	140	124	18	293	35	31	184	67	45	39	61
Femoro-popliteal lesion^21)^	3,689	2,545	1,144	41	94	1,030	3,663	3	23	2,174	800	275	415	25	457	152	75	443	175	44	91	87
Infrapopliteal-ankle lesion^21)^	2,115	1,413	702	41	84	973	2,074	17	23	1,133	480	179	305	18	256	110	48	258	157	11	93	47
Others	153	114	39	4	6	75	145	1	7	29	39	28	57	0	14	67	18	7	5	2	2	9
Total (number of regions underwent EVT)^21)^	8,257	6,027	2,230	96	199	2,149	8,116	21	98	5,353	1,562	542	747	53	873	314	153	759	337	89	207	186
Total (number of limbs underwent EVT)^22)^	7,118	5,222	1,896	80	160	1,765	6,985	20	91	4,653	1,350	464	606	45	734	268	134	634	272	76	189	169

21) When endovascular treatment performed for multiple regions, the case should be counted in each regions (If a case underwent endovascular treatment in both aorto-iliac and femoro-popliteal region, this case can be counted one in aorto-iliac, and one in femoro-popliteal region). 22) Counting the patients number not treated regions. When a case underwent endovascular treatment in multiple region, the case is counted as one case. Abbreviations; ASO: arteriosclerosis obliterans; TAO: thromboangiitis obliterans (Buerger’s disease); CAS: carotid artery stenting; CEA: carotid endarterectomy; PTA: percutaneous transluminal angioplasty; EVT: endovascular treatment; IIA: internal iliac artery

**Table table3-6:** Table 3-6 Debranch for TEVAR or EVAR

Debranch for TEVAR or EVAR	Cases
Ascending aorta-brachiocephalic-left common carotid (-left subclavian) arterial bypass	34
Right axillar-left common carotid (-left axillary) arterial bypass	284
Right common carotid-left common carotid (-left subclavian) arterial bypass	
Left common carotid-left subclavian arterial bypass or transposition	
Right axillar (subclavian)-left axillar (subclavian) arterial bypass	325
Abdominal aorta (iliac) (-celiac)-superior mesenteric-renal arterial bypass	33

Ao-F: aorto-femoral; Ax-F: axillo-femoral; F-F: femoro-femoral crossover; EVT: endovascular treatment; FPAK: femoro-popliteal (above the knee); FPBK: femoro-popliteal (below the knee); SFA: superficial femoral artery; EA: endarterectomy.

**Superficial femoral artery region**: Femoral-above-knee popliteal artery bypasses decreased by 10% from 1,810 patients in 2015 to 1,624 in 2016. The number of patients who underwent endovascular treatment increased by 460, exceeding the decrease in the number of patients who underwent bypass surgeries (**Fig. 4B**). A past history of revascularization was reported in 28% of patients. Expanded polytetrafluoroethylene (ePTFE) was used as a graft in 70% of the patients and autologous veins in 20%, with no significant differences from the case in the previous year.

**Revascularization below the knee joint**: In 2015, 726 patients underwent femoral-below the knee popliteal bypasses and 1,194 patients underwent femoral-crural/pedal artery bypasses. Furthermore, 752 patients underwent femoral-below the knee popliteal artery bypasses and 1,329 patients underwent femoral-crural/pedal artery bypasses in 2016. The number of patients who underwent femoral-crural/pedal artery bypasses increased by 11% from that in 2015 (**Fig. 4C**). The proportion of patients undergoing dialyses who underwent crural artery bypasses was 39%, unchanged from the previous year, and remained the same in 2014. This suggested that patients who underwent dialyses constituted approximately 40% of patients who had severe ischemic limbs. Autologous veins were used in 90% of the grafts.

**Thromboendarterectomy (Table 3-4)**: The number of patients who underwent thromboendarterectomy for lower limb arteries in the femoropopliteal region increased from 960 in 2015 to 1,040 in 2016 (**Fig. 4B**). The number of patients who underwent other types of surgery increased from 476 in 2015 to 546 since other items were listed as including replacements. Femoral arteriectomies with grafts were also included in this category in a large number of patients, and treatments for common femoral artery lesions difficult to deal with by endovascular treatment remained unchanged in comparison with those in the preceding year.

**Endovascular treatment (Table 3-5)**[Table table3-6]: The total number of patients undergoing endovascular treatment increased by approximately 11%, (700 patients) from 2015, and 25% of this increase was for dialysis patients. The number of endovascular therapies increased compared with the number of surgical revascularization procedures (bypass, thromboendarterectomy) and almost equaled compared with the number in 2015. Endovascular therapies for occlusive arterial disease have rapidly expanded, and surgical revascularization procedures and endovascular therapies have been equally performed. Among these, the region of the lower limb artery considerably increased from 1,803 patients in 2015 to 2,115 patients in 2016, with a rate of increase of 17%; the region of the femoropopliteal artery increased by 17%, and the region of the iliac artery increased by 6% (**Figs. 4A, 4B, 4C**).

The data were compared with those in the NCD-based Japanese nationwide registry of patients undergoing endovascular therapy (J-EVT) published on the website of the Japanese Association of Cardiovascular Intervention and Therapeutics (CVIT). Endovascular treatment of the aortic-iliac region in cardiology departments was performed in 7,587 patients in 2016. Furthermore, 5,216 patients underwent anatomical/extra-anatomical revascularization and endovascular treatment performed by vascular surgeons, constituting 40.0% of the total, which was lower than 46.0% in 2014. In 2016, 13,666 patients reportedly underwent endovascular treatment of the superficial femoral artery in J-EVT. Moreover, 5,313 patients underwent both femoral-above the knee popliteal artery bypasses and endovascular treatment by vascular surgeons, constituting 27.9% of the total, a 10% decrease from the number in 2014. In 2016, 6,943 patients underwent endovascular treatment in lower limb artery regions in J-EVT; 4,196 underwent femoral-below the knee popliteal artery bypasses, femoral-crural/pedal artery bypasses, and endovascular treatment by vascular surgeons, constituting 37.4% of the total number of patients, a 5.9% decrease from the number in 2014.

## 3. Revascularization for Acute Arterial Occlusions (Table 4)

Except for vascular trauma, 4,983 patients were reported to have acute arterial occlusions, and patients with sites in peripheral vessels below the abdominal aorta constituted approximately 80% of the total. The etiology was thrombosis in 60% and embolism in 40% of the patients. Further, 5,923 patients were classified by site of occlusion; thus, 940 patients (16%) had occlusions at multiple sites, which was almost the same as the numbers in the previous years. In addition, 74 patients underwent thrombolysis therapy, which was an additional item since 2013, with 62 patients in the previous year, indicating a slight increase. The proportion of patients overall who underwent percutaneous transluminal angioplasty (PTA)±stenting was 15%, almost the same as the number in the previous year. The rate of endovascular therapy (PTA±stenting, thrombolysis) was 26% in the abdominal aortic-iliac artery region and 15% in the femoropopliteal region, both of which were almost the same as those in the previous year.

**Table:** Table 4 Revascularization for acute arterial occlusive disease^23)^

Obstructive artery^24)^	Cases	Gender		Mortality		Etiology			Procedure						Graft materials for open surgery			
		Male	Female	30-day mortality	Hospital mortality	Embolism	Thrombosis^25)^	Others	Thrombectomy±patch^26)^	Bypass	Replacement	PTA±stent	Thrombolysis	Other	Autogenous vessel	Polyester	ePTFE	Others
Carotid artery	13	12	1	1	1	2	1	10	1	6	0	5	0	1	0	2	4	0
Subclavian artery	49	26	23	3	3	21	17	11	28	12	1	5	1	3	0	4	10	0
Axillary artery	86	46	40	3	4	31	47	8	69	10	3	7	0	7	1	3	9	0
Brachial artery	679	328	351	36	41	286	382	11	595	14	5	36	7	53	12	8	29	2
Celiac/superior mesenteric artery	120	70	50	24	30	49	34	37	53	32	2	26	5	10	23	7	5	0
Renal artery	30	20	10	2	3	6	4	20	2	7	2	21	0	1	1	4	3	0
Abdominal aorta-iliac artery	824	577	247	89	112	282	422	120	528	222	20	204	13	29	24	120	133	6
Femoro-popliteal artery	2,833	1,796	1,037	187	256	1,189	1,543	101	2,318	351	24	395	35	156	149	138	223	17
Crural artery	941	594	347	91	113	409	517	15	722	85	6	199	33	86	70	28	30	3
Pedal artery^27)^	57	35	22	2	4	22	32	3	40	10	0	14	4	5	6	3	4	0
Others	291	166	125	15	21	64	205	22	228	41	4	48	9	24	21	13	30	2
Total	4,983	3,087	1,896	362	474	1,971	2,686	326	3,791	662	59	753	74	334	247	275	417	28

23) Cases with non-traumatic acute arterial occlusion are listed in this table. Please see **Table 5-1** for acute arterial occlusion by trauma. 24) The most proximal occluded artery name is described in case whose primary occluded artery couldn’t be identified. 25) Cases with acute worsening occlusion of chronic arterial occlusive disease are excluded. Treatment for those cases are listed in **Table 3**. 26) If either thrombectomy or patch plasty is performed, cases are listed in this section. 27) Including acute occlusion of dorsalis pedis or planter artery.

The rate of artificial graft use in bypass surgery was 69% (69% in the previous year) in the femoropopliteal region and 44% (55% in the previous year) in crural arteries, indicating a slight decreasing trend in the crural artery region.

The operative/in-hospital mortality was 11%/14% (12%/15% in the previous year) for the abdominal aortic-iliac artery region, 7%/9% (9%/11% in the previous year) for the femoropopliteal arteries, 10%/12% (9%/13% in the previous year) for crural arteries, and 4%/7% (20%/27% in the previous year) for pedal arteries. These figures indicated that as usual, the prognosis was obviously poor than that for elective revascularization. The reported mortality for pedal arterial occlusions markedly increased in the previous year (2015) but clearly decreased this year. Further, 120 occlusions were reported in the celiac artery or superior mesenteric artery, and the prognosis was poor with a 20% operative mortality and 25% in-hospital mortality, with the rate of endovascular treatment being 26% (19% in the previous year), indicating a slight increase in tendency.

## 4. Treatment for Vascular Trauma (Table 5)

**Figure 5:**
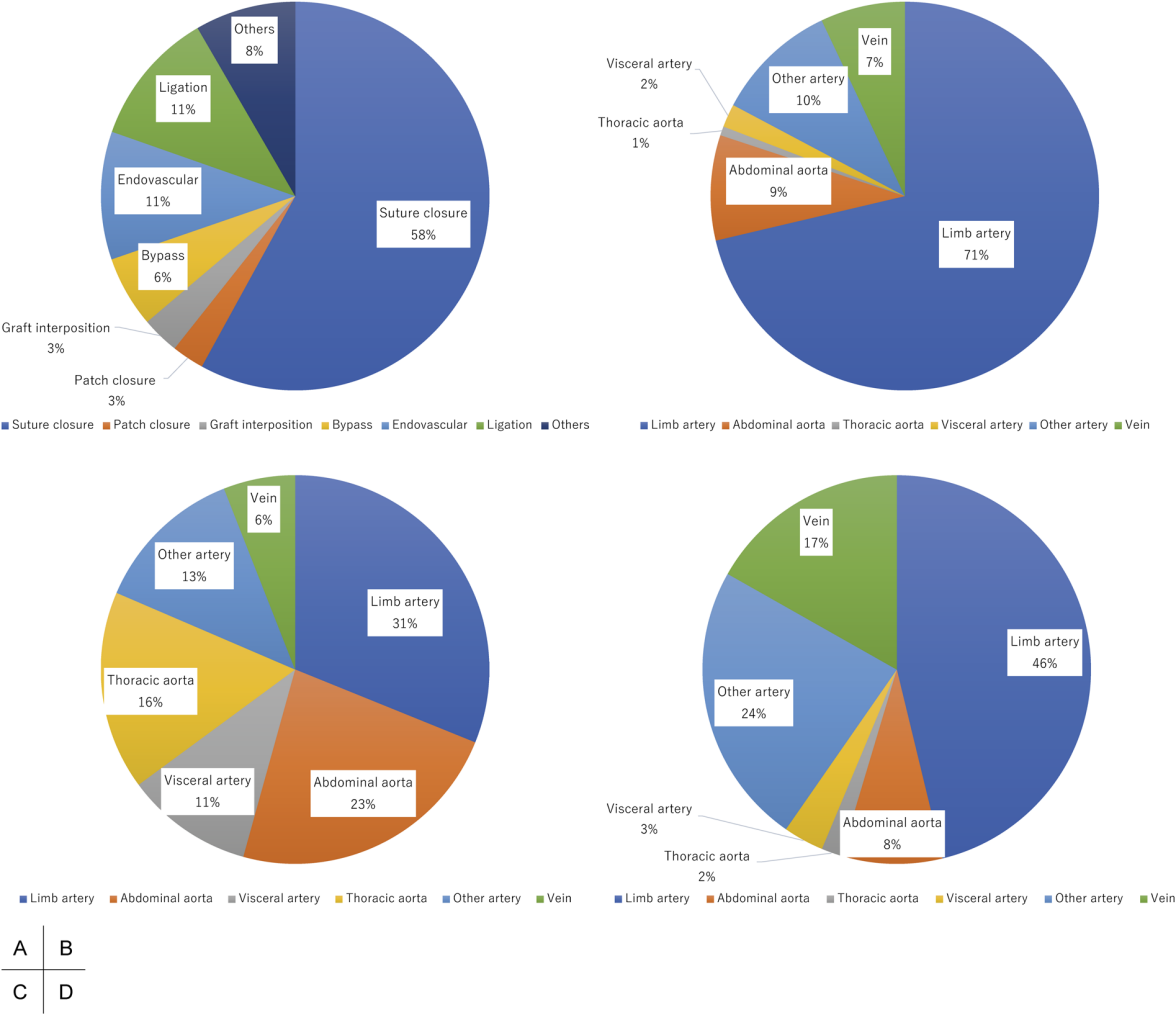
Fig. 5 Treatment procedure and location of vascular trauma in year 2016. Operation mode (**A**), location vascular trauma by iatrogenic (**B**), by traffic accident (**C**), and work-related accident (**D**).

The location of vascular trauma, cause of injury, type of surgical procedure, and type of blood vessel used as a graft in the NCD registration data in 2016 are presented in **Table 5**. Further, 2,557 patients had arterial/venous trauma. The most common cause of vascular trauma was iatrogenic in 1,831 patients (71%), followed by traffic accident in 137 (5%) and work-related in 117 (4%). The most common site of vascular injury was the arteries of the lower limbs in 1,300 patients (50%), followed by the arteries of the upper limbs in 277 patients (10%) and the abdominal-iliac arteries in 257 patients (10%). Surgical procedures were registered for 2,685 patients. Of these, 1,557 patients (58%) underwent direct suture repair, 302 patients (11%) underwent ligation, and 286 patients (10%) underwent endovascular treatment (**Fig. 5A**). Vascular grafts were used in 324 patients, and approximately 48% of the grafts were autologous.

**Table:** Table 5 Treatment for vascular trauma^28)^ Table 5-1 Arterial trauma

Injured artery	Cases	Gender		Mortality		Cause of trauma				Procedure							Status of injured artery						Prosthesis			
		Male	Female	30-day mortality	Hospital mortality	Traffic accident	Labor accident	Iatrogenic	Others	Direct closure	Patch plasty	Replacement	Bypass	Endo-vascular	Ligation	Others	Obstruction/stenosis^29)^	Bleeding without specification^30)^	GI fistula	Non-GI fistula	Pseudo-aneurysm	Others	Autogenous vessel	Polyester	ePTFE	Others
Carotid artery	40	28	12	6	10	3	1	24	12	15	1	3	5	15	1	2	3	19	1	2	6	10	3	4	1	1
Subclavian artery	51	37	14	2	3	9	1	30	11	19	2	3	5	14	4	6	9	19	0	3	8	15	1	5	4	0
Axillary artery	20	12	8	2	2	2	0	16	2	9	3	2	2	5	2	2	3	7	0	1	8	3	2	2	2	0
Brachial artery	321	206	115	8	13	4	18	264	35	241	3	5	12	12	37	21	25	57	0	5	199	45	16	1	4	0
Descending aorta (thoracic/thoracoabdominal)	60	49	11	10	11	25	2	14	19	10	0	2	2	35	3	11	4	27	3	8	17	5	0	2	2	0
Celiac/superior mesenteric artery	54	39	15	4	5	10	3	31	10	16	0	0	6	25	8	2	8	26	4	1	10	6	3	0	2	0
Renal artery	15	11	4	3	3	6	1	5	3	2	1	1	1	11	1	1	1	11	0	2	0	1	0	2	2	0
Abdominal aorta-iliac artery	257	128	129	29	38	35	10	163	49	58	9	30	38	116	11	12	43	127	10	11	26	49	7	40	31	3
Femoro-popliteal artery	1,246	798	448	169	208	23	28	991	204	944	45	32	73	36	82	85	126	274	0	20	410	459	92	19	36	6
Crural artery	54	40	14	2	2	9	8	27	10	24	2	3	12	4	9	3	16	17	1	0	16	6	16	1	1	0
Others	292	199	93	27	31	16	27	166	83	129	4	4	8	29	92	45	30	141	2	9	55	67	11	1	1	3
Total	2,357	1,513	844	252	316	128	97	1,702	430	1,453	67	78	150	284	249	186	252	705	21	60	746	656	145	69	76	12

28) Iatrogenic pseudoaneurysm in endovascular treatment is listed in **Table 5-1**. 29) Including arterial dissection. 30) Without GI fistula or non-GI fistula. Cases with vessel injury involving both vein and accompanying artery are listed in **Table 5-1**. Abbreviation; GI: gastro-intestinal

**Table:** Table 5-2 Venous trauma

Injured veins	Cases	Cause of trauma				Procedure							Prosthesis			
		Traffic accident	Labor accident	Iatrogenic	Other	Direct closure	Patch plasty	Replacement	Bypass	Endo-vascular	Ligation	Others	Autogenous vessel	Polyester	ePTFE	Others
Superior vena cava	3	0	0	2	1	1	0	0	2	0	0	1	0	0	2	0
Inferior vena cava	20	2	0	12	6	13	2	0	0	1	2	3	1	0	1	0
Brachiocephalic-subclavian vein	7	0	0	6	1	4	0	0	1	0	1	1	0	0	1	0
Iliac-femoral-popliteal vein	69	1	4	50	14	47	3	3	5	0	10	9	8	1	4	0
Others	104	6	16	61	21	41	0	3	3	1	40	25	2	0	3	1
Total	200	9	20	129	42	104	5	6	9	2	53	39	11	1	9	1

### 1) Iatrogenic vascular trauma (Fig. 5B)

Looking at 1,862 sites of iatrogenic vascular trauma in 1,831 patients by site, arteries of the lower limbs constituted the highest proportion (1,018 patients, 54%). This was followed by arteries of the upper limbs (310 patients, 16%), constituting 71% of the combined upper and lower limbs. Most of these lesions were considered to be complications of intravascular catheter examinations or puncture site complications associated with treatment.

### 2) Traffic accidents (Fig. 5C)

The most common site from traffic accidents in 137 patients (151 sites) was the upper or lower limb arteries in 47 patients (31%). The second most common was the abdominal aorta and iliac arteries (35 patients, 23%), followed by the descending aorta and thoracoabdominal aorta (25 patients, 16%) and the visceral arteries (16 patients, 10%). Blood vessels in the limbs close to the body surface are susceptible to direct external force and injury; however, unlike other causes, thoracic and abdominal aortic injuries in regions protected by the thorax and abdominal wall are common in traffic accidents. This is considered to be due to high-energy trauma with rapid deceleration such as collision.

### 3) Work-related (Fig. 5D)

Accidents at work such as falls from high places and machinery-related injuries were assumed, and 119 lesions in 117 patients were registered. As expected, arteries in the limbs, which are also close to the body surface and subject to external force, constituted 63% (75 patients).

### 4) Summary

Vascular traumas registered in the 2016 NCD database were reviewed. Although the number of registered patients slightly increased compared with the number in 2015, no major differences were observed in the cause of trauma, site of trauma, type of vascular graft, and treatment method.

## 5. Surgery for Vascular Complications after Revascularization (Table 6)

The number of patients registered with sites in the thoracic to thoracoabdominal aortic region was as low as those reported up to 2014, and the number of revascularization complications in this region could not be evaluated.

### 1) Vascular graft infections (Table 6-1)

As vascular graft infection, 526 cases were registered. 50.4% of which were the other region, including the arch branching and upper limb artery. In this region, the most prevalent condition of infection is the cutaneous fistula of vascular grafts. Many of which were inferred to be infection in the shunts for dialysis. 25.5% of graft infection were femoro-distal artery. The overall operative mortality was 8.4%, and in-hospital mortality was 14.3%.

**Table:** Table 6 Revascularization for vascular complication after revascularization Table 6-1 Graft infection

Position of infected graft	Cases	Mortality		Status of infected graft				Procedure for graft infection			Material for revision or redo surgery				
		30-day mortality	Hospital mortality	Sepsis	Graft-GI fistula^32)^	Graft-skin fistula^32)^	Others	In-situ replacement	Extra-anatomical bypass	Others	Polyester	ePTFE	Autogenous vessel	Cryo-preserved homograft	Others
Descending thoracic aorta	4	0	3	2	1	0	1	1	0	2	2	0	0	0	0
Thoracoabdominal aorta	7	0	1	3	2	2	0	4	0	1	5	1	0	0	0
Abdominal aorta-iliac artery	74	9	23	29	31	3	23	29	0	31	33	12	4	0	3
Abdominal aorta-femoral artery	42	3	5	9	7	10	19	9	0	19	12	7	9	0	0
Femoro-distal artery	134	18	23	43	1	53	44	25	0	89	10	34	30	0	7
Others^31)^	265	14	20	57	5	91	124	23	0	209	23	61	26	0	10
Total	526	44	75	143	47	159	211	91	0	351	85	115	69	0	20

31) Cases with graft infection involving aortic arch branch or upper limb artery are listed on this column. 32) Including anastomotic disruption. Abbreviation; GI: gastrointestinal

### 2) Anastomotic aneurysms (noninfectious) (Table 6-2)

Further, 144 patients with anastomotic aneurysms were registered. By region, the femoral artery was the most common, followed by the abdominal aorta and axillary-upper limb arteries, which were similar to those in 2015. Femoral arteries were mainly repaired by replacement, and abdominal aortas were repaired by stent graft endovascular repair.

**Table:** Table 6-2 Anastomotic aneurysm^33)^

Location of anastomotic aneurysm	Cases	Mortality	Cause of aneurysm treated at the primary operation					Repair procedure				Material for repair surgery			
		30-day mortality	Degenerative	Takayasu arteritis^34)^	Other vasculitis^35)^	Infection	Others	Replacement	Exclusion and bypass	Stent graft	Others	Polyester	ePTFE	Autogenous vessel	Others
Aortic arch branch	10	0	3	0	0	0	7	1	0	4	5	0	3	1	1
Upper limb artery including axillar artery	24	0	7	0	0	2	15	3	2	1	18	1	7	4	0
Thoracic aorta	12	1	1	0	0	1	10	3	1	2	6	2	1	0	2
Splanchnic artery	1	0	0	1	0	0	0	1	0	0	0	0	1	1	0
Renal artery	0	0	0	0	0	0	0	0	0	0	0	0	0	0	0
Abdominal aorta	25	1	17	0	0	1	7	4	0	17	4	16	2	0	1
Iliac artery	15	1	7	1	0	1	6	5	0	5	5	7	5	1	2
Femoral artery	46	1	24	1	0	4	17	16	2	0	29	10	10	2	3
Popliteal or more distal lower limb artery	15	1	7	0	0	2	6	4	2	0	9	0	4	5	1
Total	144	5	64	2	0	10	68	35	7	28	75	35	31	13	10

33) Cases with infected pseudoaneurysm located at the anastomotic site to the artificial graft are listed in **Table 6-1**. 34) Including the atherosclerotic aneurysm. 35) Including TAO, collagen disease, Behcet disease, and fibromuscular dysplasia.

### 3) Autologous vascular graft aneurysms (Table 6-3)

Autologous vascular graft aneurysms were registered in upper limb arteries in 23 patients and lower limb arteries in 32 patients, but not in the abdominal splanchnic arteries.

**Table:** Table 6-3 Autogenous graft aneurysm

Revascularization area	Cases	Mortality	Repair procedure		
		30-day mortality	Replacement	Bypass	Others
Visceral artery	0	0	0	0	0
Upper limb artery	23	0	5	3	15
Lower limb artery	32	0	11	8	14
Others	11	0	1	2	8
Total	66	0	17	13	37

### 4) Vascular graft deterioration (Table 6-4)

The number of patients for whom operations for artificial vascular graft deterioration were reported was 110. There were 52 in 2014 and 97 in 2015, indicating increases. The number of reports by initial surgical procedure (2014→2015→2016) was 19 patients undergoing replacement→29 patients→40 patients, 19 patients undergoing bypass surgeries→46 patients→49 patients, and 3 patients undergoing stent grafting→6→12 patients. Although deterioration of artificial blood vessels was reported for both polyester and ePTFE, the rate of deterioration could not be calculated because the population parameter was unknown.

**Table:** Table 6-4 Graft degeneration

Revascularization	Cases	Mortality	Initial revascularization procedure				Degenerative material			Repair procedure					Graft material		
		30-day mortality	Replacement	Bypass	Stent graft	Others	Polyester	ePTFE	Others	Replacement	Bypass	Stent graft	Patch plasty	Others	Polyester	ePTFE	Others
Descending thoracic aorta	3	0	2	1	1	0	2	1	0	0	1	1	0	1	1	1	0
Thoracoabdominal aorta	4	1	3	1	1	0	3	0	1	1	1	2	0	1	1	1	1
Abdominal aorta-femoral artery	20	0	7	10	3	1	10	7	3	2	9	3	1	5	5	10	1
Femoro-popliteal artery	20	1	2	15	0	5	5	16	1	4	7	0	1	9	3	9	6
Others	66	2	28	24	7	11	14	32	21	12	24	3	1	31	5	26	16
Total	110	4	40	49	12	17	32	55	25	19	40	8	3	47	15	46	23

## 6. Venous Surgery (Table 7)

### 1) Varicose veins of lower limbs (Table 7-1)

**Figure 6:**
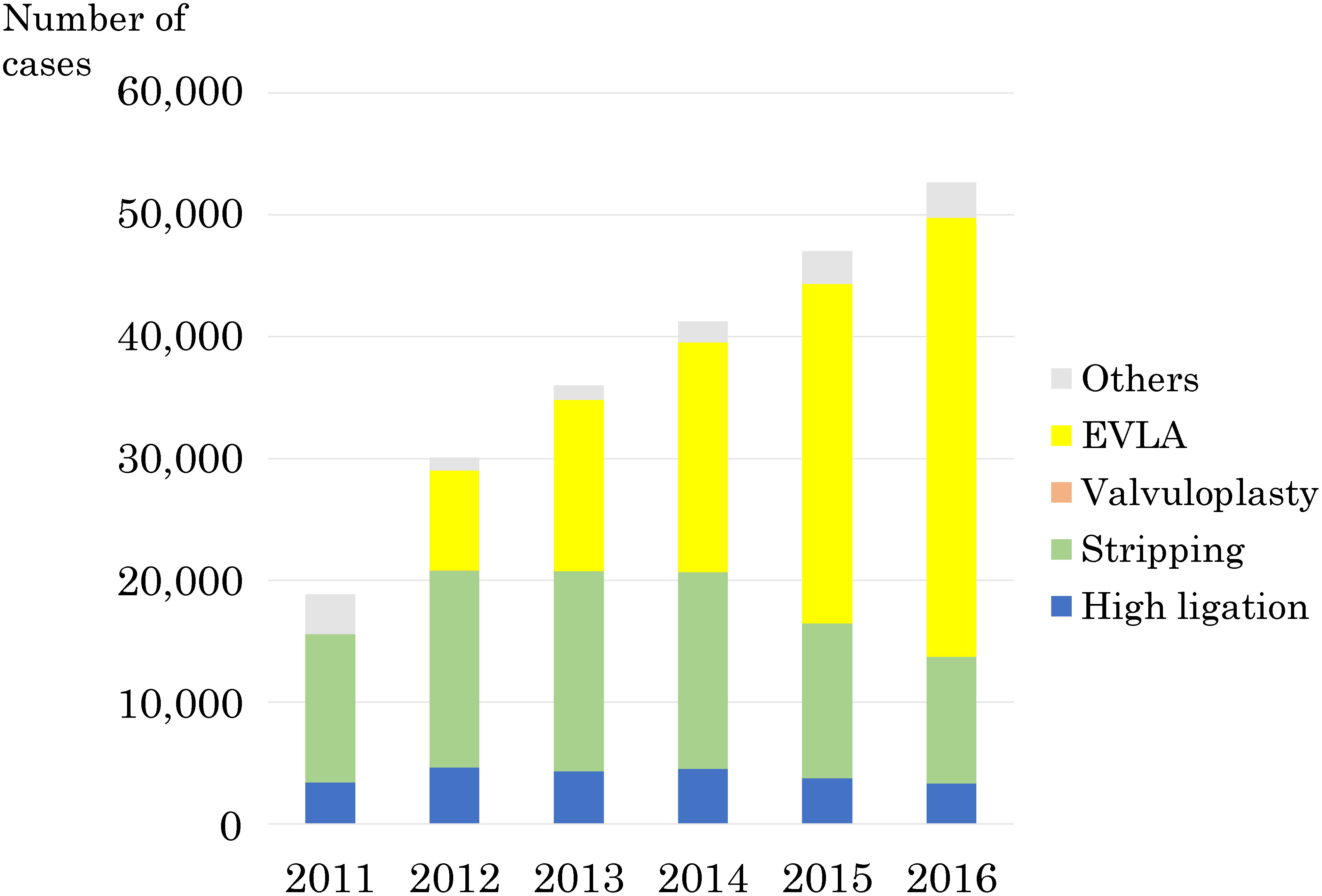
Fig. 6 Changes of varicose veins treatment in year 2011, 2012, 2013, 2014, 2015 and 2016.

The number of varicose vein surgeries of the lower limbs has been increasing since 2011, with a highest record of 52,639 patients in 2016. Stripping procedures (± sclerotherapy) and high ligation procedures were less frequent, and endovenous laser ablation (EVLA) (± sclerotherapy) increased to 36,036 patients (68.5%) (**Fig. 6**). In Japan, 1,470 nm lasers and radiofrequency catheters were covered by health insurance in 2014. In the present record, radiofrequency ablation may have been registered in other procedures. Nonetheless, endovascular ablation was performed in more patients and was the first-line treatment for varicose veins of lower limbs.^10)^

**Table:** Table 7 Venous surgery Table 7-1 Varicose veins

Varicose veins treatment	Cases^36)^	Male	Female	30-day mortality
High ligation±sclerotherapy	3,334	1,112	2,222	0
Stripping±sclerotherapy	10,397	4,098	6,299	0
Valvuloplasty	1	1	0	0
EVLA±sclerotherapy^37)^	36,036	12,611	23,425	0
Others	2,871	799	2,072	0
Total	52,639	18,621	34,018	0

36) Only one procedure can be registered in one leg. 37) EVLA: endovenous laser ablation

EVLA: endovenous laser ablation.

### 2) Deep vein thrombosis of the lower limbs (including deep vein stenosis and occlusion) (Table 7-2)

A total of 469 patients who underwent surgery for deep vein thrombosis (DVT) of the lower limbs were registered. Majority, 267 patients (56.9%), underwent inferior vena cava filter placement, and 135 patients (28.8%) underwent filter removal. The rate of filter placement slightly decreased compared with 58.6% in 2015. Endovascular treatment of catheter-directed thrombolysis (CDT) was performed in 37 patients (7.9%), and endovascular treatment for stenosis was performed in 20 patients (4.3%), the same as that in the previous year; however, thrombectomy decreased from 64 patients in 2015 to 37 patients (7.9%).

**Table:** Table 7-2 Deep vein thrombosis (including venous stenosis or obstruction)

Deep vein thrombosis treatment	Cases	Male	Female	30-day mortality
Thrombectomy	41	20	21	0
Catheter-directed thrombolysis^38)^	37	16	21	0
Bypass (peripheral venous reconstruction)	3	1	2	0
IVC filter insertion^39)^	267	112	155	2
IVC filter retrieval^39)^	135	59	76	0
Direct surgery of stenosis^40)^	6	1	5	0
Endoluminal treatment of stenosis	20	12	8	2
Others	6	5	1	0
Total	469	207	262	4

38) Including the catheter-directed thrombolysis using hydrodynamic thrombectomy catheter. 39) Including temporary IVC filter. 40) Including obstruction.

### 3) Upper limb and cervical vein stenosis and occlusion (Table 7-3)

The number of surgeries reported was 148, a slight increase from the number in 2015. Endovascular treatment was mostly performed to resolve venous stenosis in 87 patients (58.8%).

**Table:** Table 7-3 Upper limb vein stenosis or obstruction

Treatment of vein stenosis (obstruction)	Cases	Male	Female	30-day mortality
Thrombectomy	44	25	19	0
Catheter-directed thrombolysis^41)^	4	3	1	0
Bypass	9	6	3	0
SVC filter insertion^42)^	2	2	0	0
Direct surgery of stenosis	5	2	3	0
Endoluminal treatment of stenosis	87	55	32	1
Others	14	9	5	0
Total	148	91	57	1

41) Including the catheter-directed thrombolysis using hydrodynamic thrombectomy catheter. 42) Including temporary IVC filter.

### 4) Vena cava reconstruction (Table 7-4)

The number of patients undergoing surgeries decreased to 67 patients from 75 in 2015, and the proportion of venous reconstruction surgeries for the inferior vena cava to that for the superior vena cava was similar to that in 2015. The most common etiology was tumors in 57 patients (85.1%), and only 1 operative and in-hospital death were reported each (1.5%). Surgical results were more favorable than those in 2015.

**Table:** Table 7-4 Vena cava reconstruction

Vena cava reconstruction	Cases	Mortality		Etiology			Treatment procedures					Material for open surgery			
		30-day mortality	Hospital mortality	Tumor	Thrombus	Others	Patch plasty	Bypass	Replacement	PTA±stent	Others	Autogenous vessel	Polyester	ePTFE	Others
SVC reconstruction	16	0	1	11	3	2	3	6	5	3	0	0	0	11	1
IVC reconstruction	51	0	0	46	1	4	12	1	10	2	26	10	3	9	2
Total	67	0	1	57	4	6	15	7	15	5	26	10	3	20	3

Abbreviations; IVC: inferior vena cava; SVC: superior vena cava

### 5) Budd-Chiari syndrome (Table 7-5)

Only 1 patient each underwent shunt surgery and percutaneous shunt creation, and the number of registered patients was extremely small as in 2015.

**Table:** Table 7-5 Budd-Chiari syndrome

Treatment	Cases	Gender		Mortality		Material for open surgery			
		Male	Female	30-day mortality	Hospital mortality	Polyester	ePTFE	Autogenous vessel	Others
Shunting	1	0	1	0	0	0	0	0	0
Percutaneous shunting	1	1	0	0	0	0	0	0	1
Surgical recanalization	0	0	0	0	0	0	0	0	0
Total	2	1	1	0	0	0	0	0	1

### 6) Other (Table 7-6)

There were 17 patients with deep vein aneurysm plication and suturing, and 3 patients underwent splanchnic vein aneurysm surgeries, indicating that the disease was rare as was the case in 2015.

**Table:** Table 7-6 Other surgery

Treatment	Cases	Gender		Mortality		Material for open surgery			
		Male	Female	30-day mortality	Hospital mortality	Polyester	ePTFE	Autogenous vessel	Others
Plication of deep venous aneurysm^43)^	17	8	9	1	0	0	0	0	0
Plication of abdominal venous aneurysm	3	0	3	1	1	0	0	0	0
Others	1,117	552	565	52	95	0	0	0	0
Total	1,137	560	577	54	96	0	0	0	0

43) Including patch plasty.

## 7. Other Vascular Diseases and Related Surgeries (Table 8)

Only vascular access surgery significantly increased in 2016 compared with that in 2015.

### 1) Popliteal artery entrapment syndrome and cystic adventitial disease (CAD) (Table 8-1 and 8-2)

Although these were rare diseases from the start, the number of CAD did not change in 2016 compared with that in 2015, but that of popliteal artery entrapment syndrome decreased significantly.

**Table:** Table 8 Other vascular diseases Table 8-1 Popliteal artery entrapment syndrome

Treatment	Cases	30-day mortality
Myotomy	9	0
Revascularization	19	0
Total	21	0

**Table:** Table 8-2 Adventitial cystic disease

Treatment	Cases	30-day mortality
Cyst excision±patch plastry	22	1
Replacement	13	0
Bypass	5	0
Total	37	1

### 2) Thoracic outlet syndrome (Table 8-3)

The number has remained almost the same over the past several years, and the disease continues to be rare.

**Table:** Table 8-3 Throracic outlet syndrome (TOS)

Treatment	Cases	Male	Female	30-day mortality	Type of TOS^44)^		
					Neurogenic	Venous	Arterial
Rib resection^45)^	2	0	2	0	2	1	0
Rib resection+scalenectomy	12	9	3	0	5	2	6
Bypass	4	4	0	0	1	0	4
Total	14	9	5	0	7	3	6

44) In the case with mixture type, the type having the most significant impact on the clinical symptom is listed. But, if the impacts are similar, multiple response is allowed. 45) Including cervical rib.

### 3) Vascular access surgery (Table 8-4)

Although there is a tendency for increases every year, the speed of increase is accelerating, with an increase to 4,700 patients from the last year.

**Table:** Table 8-4 Vascular access operation

Treatment	Cases	30-day mortality
Arteriovenous access creation by autogenous material	14,533	134
Arteriovenous access creation by artificial material^46)^	3,320	42
Open surgery for access repair	2,801	34
Endovascular access repair	11,120	46
Arterial transposition	503	11
Arteriovenous access aneurysm repair	502	7
Total	32,779	274

46) including cases with access repair using artificial graft.

### 4) Surgical treatment for lymphedema (Table 8-5)

Although the number of surgeries significantly decreased from the previous year, there seems to be large fluctuations in the numbers.

**Table:** Table 8-5 Surgery for lymphedema

Treatment	Cases	Male	Female	30-day mortality
Lymphovenous anastomosis	0	0	0	0
Lymph drainage operation	3	2	1	0
Resection	36	23	13	0
Total	39	25	14	0

### 5) Sympathectomy (Table 8-6)

In 2016, the number of patients was 33, with the level remaining almost unchanged.

**Table:** Table 8-6 Sympathectomy

Sympathectomy	Cases	30-day mortality
Thoracic sympathectomy	23	0
Lumbar sympathectomy	10	0
Total	33	0

### 6) Upper limb and lower limb amputation (Table 8-7 and 8-8)

Upper limb amputation remained unchanged this year; however, leg amputations showed a decreasing trend from an increasing trend, and there is a need to observe the future course.

**Table:** Table 8-7 Amputation of upper limb

Amputation level	Cases	30-day mortality
Digit	17	0
Forearm/upper arm	2	0
Total	19	0

**Table:** Table 8-8 Amputation of lower limb^47)^

Amputation level	Cases	30-day mortality	Etiology			
			ASO	DM-ASO	TAO	Others
Toe	564	12	236	271	2	55
Transmetatarsal	247	3	77	141	3	26
Lisfranc/Chopart	40	3	12	22	0	6
Syme	6	1	2	4	0	0
Below-Knee	257	14	116	126	1	14
Through-Knee/Above-Knee	295	25	167	87	2	39
Hip	2	0	1	0	1	0
Total	1,411	58	611	651	9	140

47) Amputations not due to ischemia are not included. Abbreviations; ASO: arteriosclerosis obliterans; DM-ASO: diabetic ASO; TAO: thromboangiitis obliterans (Buerger’s disease)

## Conclusion

Annual reports of vascular surgery have been released every year since 2011, when the registration into NCD started, and an overall view of vascular surgeries in 2016 was clarified. This article provides a glimpse into the current status of vascular surgeries in Japan, in which details regarding surgeries are changing over time.

One of the major purposes of participating in the NCD is to improve the quality of medical care using NCD data. Limiting the data entry to essential items is essential to facilitate the entry of data considering the busy schedule of medical care. Despite this, the number of items to enter in order to improve the evaluation of the quality of medical care is increasing every year. Fortunately, the rate of mortality is too low for vascular surgeries, except for major vascular surgeries to be used as an evaluation index. The future goal is to add a function into the NCD to allow the comparison of quality of risk-adjusted vascular surgical practice at each institution with national standards. In 2018, the JSVS started a nationwide multicenter observational study of treatment options between open surgery and endovascular stent grafting for ruptured abdominal aortic aneurysms. As a model study, a retrospective study of treatment and prognosis for infected abdominal aortic aneurysm and common iliac artery aneurysm was conducted. Furthermore, in 2019, a retrospective study of surgical procedures and prognosis for popliteal artery entrapment syndrome was conducted to address these issues. In particular, the retrospective study on the treatment and prognosis of infected abdominal aortic aneurysms and common iliac artery aneurysm was accepted for publication in the British Journal of Surgery and will be published. In addition, a public offering of new research topics in the field of vascular surgery using NCD data has been started. Since 2018, an analysis of factors affecting surgical methods and prognoses for infection of artificial vascular/stent grafts in the abdominal aortic region; an analysis of the effects of malignant neoplasms on the prognoses of patients with arteriosclerosis obliterans and severe lower limb ischemia; and an analysis of the onset of wound complications after bypass surgery for ischemic limbs have been started. In 2019, a multicenter observational study on medical institution cooperation in emergency care for aortic and peripheral artery emergency care and the results of bypass surgery for collagen disease- and vasculitis-related critical limb ischemia in Japan has also been started. Site visits were started in 2018 to improve the reliability of the data. Due to the COVID-19 pandemic in 2020, remote audits over the web are being considered.

The aim is to continue developing the vascular surgery database on NCD with the members, and the sincere hope is that this database will help provide high-quality medical care to patients suffering from vascular diseases.

## Appendix

Team responsible for analyzing the 2014 annual report as follows; Database Management Committee of the Japanese Society for Vascular Surgery: Nobuya Zempo (Chairman), Nobuyoshi Azuma (Vice-chairman), Yukio Obitsu (Vice-chairman), Yoshinori Inoue, Jin Okazaki, Hideaki Obara, Hirono Satokawa, Kunihiro Shigematsu, Ikuo Sugimoto, Hiroshi Banno, Naoki Fujimura, Akihiro Hosaka, Shinsuke Mii, Noriyasu Morikage, Terutoshi Yamaoka, Tetsuro Miyata (Observer), Kimihiro Komori (Chief director of the Japanese Society for Vascular Surgery)

NCD Vascular Surgery Data analyzers: Arata Takahashi

## Notes

This is a translation of Jpn J Vasc Surg 2021; 30: 23–41.
